# 肺鳞癌*EGFR*与*KRAS*基因突变状态分析

**DOI:** 10.3779/j.issn.1009-3419.2015.10.04

**Published:** 2015-10-20

**Authors:** 卉 张, 新杰 杨, 娜 秦, 曦 李, 惠夷 杨, 靖颖 农, 嘉林 吕, 羽华 吴, 权 张, 新勇 张, 敬慧 王, 丹 苏, 树才 张

**Affiliations:** 1 101149 北京, 首都医科大学附属北京胸科医院/北京市结核病胸部肿瘤研究所肿瘤内科 Department of Medical Oncology, Beijing Chest Hospital, Capital Medical University/Beijing Tuberculosis and Thoracic Tumor Research Institute, Beijing 101149, China; 2 510663 广州, 益善医学检验所 SurExam Clinical Testing Centre, Guangzhou 510663, China; 3 101149 北京, 首都医科大学附属北京胸科医院/北京市结核病胸部肿瘤研究所病理科 Department of Pathology, Beijing Chest Hospital, Capital Medical University/Beijing Tuberculosis and Thoracic Tumor Research Institute, Beijing 101149, China

**Keywords:** 肺肿瘤, 表皮生长因子受体, *KRAS*基因, 突变, 鳞癌, Lung neoplasms, Epidermal growth factor receptor, *KRAS* gene, Mutation, Squamous

## Abstract

**背景与目的:**

表皮生长因子受体(epidermal growth factor receptor, *EGFR*)突变和*KRAS*基因突变是非小细胞肺癌(non-small cell lung cancer, NSCLC)靶向治疗的重要分子标志, 但关于肺鳞癌中*EGFR*和*KRAS*基因突变情况的报道甚少。本研究旨在分析肺鳞癌*EGFR*和*KRAS*基因突变与临床特征的关系。

**方法:**

收集初治肺鳞癌患者139例, 有可供检测的肿瘤组织标本。利用突变富集液相芯片法进行*EGFR*和*KRAS*基因突变检测。

**结果:**

139例肺鳞癌中, *EGFR*基因突变25例(18%), *KRAS*基因突变7例(5%), *EGFR*和*KRAS*基因同时发生突变1例(0.7%)。女性和不吸烟患者*EGFR*基因突变率高于男性和吸烟患者(33.3% *vs* 16.5%, 29.6% *vs* 16.1%), 但差异均无统计学意义(*P* > 0.05, *P* > 0.05);不同年龄、分期及病理取材标本之间差异均无统计学意义(*P* > 0.05)。男性患者*KRAS*基因突变率高于女性患者(5.5% *vs* 0%), 但差异无统计学意义(*P* > 0.05);在不同年龄、分期、病理取材标本及是否吸烟各亚组分析中*KRAS*基因突变差异无统计学意义(*P* > 0.05)。

**结论:**

肺鳞癌患者*EGFR*和*KRAS*基因突变发生率均较低, 且都与临床特征无明显相关。肺鳞癌患者使用酪氨酸激酶抑制剂(tyrosine kinase inhibitors, TKIs)靶向治疗药物之前, 也应检测*EGFR*基因和*KRAS*基因的突变情况。

## Abstract (en)

**Background and objective:**

Activating mutations in epidermal growth factor receptor (EGFR) and KRAS are important markers in non-small cell lung cancer.However, *EGFR* and *KRAS* gene mutations in lung squamous cell carcinoma are rarely reported.The aim of this study was to analyze *EGFR* and *KRAS* gene mutation rate and their relationship with clinical features in patients with lung squamous cell carcinomas.

**Methods:**

A total of 139 patients undergoing treatment for na?ve lung squamous cell carcinomas with tumor tissue samples available for testing were recruited.*EGFR* and *KRAS* mutation statuses of the tumor samples were detected using a mutant enriched liquid chip.

**Results:**

Of the 139 cases of lung squamous cell carcinoma, *EGFR* mutations were detected in 25 cases (18%), *KRAS* mutations were detected in 7 cases (5%), and the presence of both *EGFR* and *KRAS* mutations was detected in 1 case (0.7%).*EGFR* mutations occurred more often in females than in males (33.3% *vs* 16.5%) and in patients that never smoked than in those who smoke (29.6% *vs* 16.1%).However, the difference did not reach statistical significance (*P* > 0.05).No significant differences were observed in age, stage, and different biopsy type.*KRAS* mutations occurred more often in males than in females (5.5% *vs* 0%), but the difference did not reach statistical significance (*P* > 0.05).No significant differences were observed in age, stage, different biopsy type, and smoking status (*P* > 0.05).

**Conclusion:**

*EGFR* and *KRAS* mutations were low in lung squamous cell carcinomas, and had no significant correlation with clinical features.Before using tyrosine kinase inhibitor targeted therapy, *EGFR* and *KRAS* mutations should be detected in patients with lung squamous cell carcinomas.

目前已有多项大型Ⅲ期临床研究^[1-3]^结果证实存在表皮生长因子受体(epidermal growth factor receptor, *EGFR*)突变的非小细胞肺癌(non-small cell lung cancer, NSCLC)患者一线应用EGFR酪氨酸激酶抑制剂(tyrosine kinase inhibitors, TKIs)具有较好的临床疗效和安全性, 而KRAS基因突变也与肺癌治疗的耐药相关。但研究也发现TKI药物治疗更多的是给肺腺癌患者带来巨大的临床获益, 而占NSCLC 20%-30%的肺鳞癌却仍无效的靶向药物指导临床实践^[4]^, 究其原因可能是由于缺乏对其生物学特征的了解。*EGFR*基因突变的肺鳞癌患者是否也具有肺腺癌突变者同样的靶向治疗疗效, 目前还缺乏大样本的前瞻性研究。本研究通过检测分析肺鳞癌患者肿瘤组织标本*EGFR*和*KRAS*基因的突变状况及与临床特征之间的关系, 以期为临床指导患者进行有针对性的治疗提供依据。

## 1 资料与方法

### 1.1 资料

收集首都医科大学附属北京胸科医院2006年1月7日-2014年3月21日收治的初治肺癌患者139例, 所有患者均经病理组织学免疫组化证实为肺鳞癌并有可供检测的肿瘤组织标本。其中男性127例, 女性12例。年龄33岁-82岁; 吸烟(吸烟指数 > 20包/年)93例, 轻度吸烟(0 < 吸烟指数 < 20包/年)19例, 不吸烟(吸烟指数为0)27例; 按2002年美国癌症研究联合会(American Joint Committee on Cancer, AJCC)癌症分期手册(第6版)NSCLC分期标准, Ⅰ期25例, Ⅱ期27例, Ⅲ期38例, Ⅳ期49例。

### 1.2 主要仪器与试剂

DNA提取试剂盒(美国Promega公司); 引物、探针合成(广州英韦创津生物科技有限公司); 蛋白酶K(德国Merck公司); 链霉亲和素-藻红蛋白、Taq酶和dNTP(美国Invitrogen公司); DNA聚合酶(美国Promega公司); 微球和Luminex 200^TM^液相芯片阅读仪(美国Luminex公司); PCR仪(美国BIO-RAD公司); Nanodrop 1000分光光度计(美国Thermo Scientific公司); 低温离心机(德国Eppendorf公司)。

### 1.3 突变富集液相芯片法

切取5 μm石蜡包埋标本10张, 用DNA提取试剂盒从样本中抽提纯化DNA(具体参照产品说明书), 随后用Nanodrop 1000分光光度计对DNA浓度进行定量; 所有样品所提的DNA的量足以用于检测。取纯化后的DNA作为模板进行PCR预扩增, 多重PCR反应条件为, 94 ℃ 30 s, 56 ℃ 30 s, 72 ℃ 30 s, 共35个循环; 预扩增产物进行酶切反应, 特异切除野生型, 反应条件为37 ℃ 30 min; 取酶切产物作为模板进行PCR扩增富集突变型, 在96 ℃条件下孵育2 min, 反应条件为94 ℃ 30 s, 52 ℃ 1 min, 74 ℃ 2 min, 共40个循环; 第二次PCR产物与交联了特异探针的微球杂交, 杂交反应在96孔板上进行, 将PCR产物、微球混合液和杂交液加入反应管中, 95 ℃ 5 min, 然后60 ℃杂交15 min, 加入链霉亲和素-藻红蛋白, 60 ℃反应5 min; 于Luminex阅读仪上读取数据。突变分析由益善医学检验所完成。

### 1.4 统计学方法

采用SPSS 21.0统计软件进行统计分析, *EGFR*与*KRAS*基因突变状态和临床特征分析采用卡方检验和*Fisher’s*精确检验。以*P* < 0.05为差异有统计学意义。

## 2 结果

### 2.1 突变检测结果

139例肺鳞癌*EGFR*基因突变检测中, 18外显子G719X突变3例(2.2%); 19外显子缺失突变9例(6.5%); 20外显子突变6例(4.3%); 21外显子突变6例(4.3%); 同时存在两种突变1例(0.7%); 野生型114例, (82.0%)。*KRAS*基因突变检测中, 外显子2突变6例(4.3%); 外显子3突变1例(0.7%); 野生型132例(95.0%)。同时存在*EGFR* 19外显子缺失突变合并*KARS*外显子突变1例(0.7%)(表 1)。

**1:** *EGFR*和*KRAS*基因突变检测结果 Results of *EGFR* and *KRAS* mutation status

	*n*=139	%
*EGFR* gene		
Exton 18 (G719X)	3	2.2
Exton 19 (19del)	9	6.5
Exton 21	6	4.3
L858R	5	3.6
L861Q	1	0.7
Exton 20	6	4.3
T790M	4	2.9
H337_V774ins H	1	0.7
S768I	1	0.7
Combination of two mutations	1	0.7
19del+20T790M	1	0.7
Wild type	114	82.0
*KRAS* gene		
Mutation	7	5.0
Exton 2	6	4.3
Exton 3	1	0.7
Wild type	132	95.0
Combination of *EGFR* and *KRAS* mutations	1	0.7
EGFR:epidermal growth factor receptor.		

### 2.2 突变与临床特征的关系

女性和不吸烟的患者*EGFR*基因突变率高于男性和吸烟患者(33.3% *vs* 16.5%, 29.6% *vs* 16.1%), 但差异无统计学意义(*P* > 0.05, *P* > 0.05);不同年龄、分期及病理取材标本之间差异无统计学意义(*P* > 0.05)。与*EGFR*基因突变不同的是, 男性*KRAS*基因突变率高于女性(5.5% *vs* 0), 但差异无统计学意义(*P* > 0.05);年龄、分期、吸烟史及病理取材方式各亚组分析中均未发现差异有统计学意义(*P* > 0.05)(表 2)。

**2:** *EGFR*与*KRAS*基因突变亚组分析 Subgroup analysis of *EGFR* and *KRAS* mutation status

Clinical characteristic	*n* (%)	*EGFR* gene		*P*	*KRAS* gene *n*=139 (%)		*P*
		Mutation (%)	Wild type (%)		Mutation (%)	Wild type (%)	
Gender				0.228			> 0.999
Male	127 (91.4)	21 (16.5)	106 (83.5)		7 (5.5)	120 (94.5)	
Female	12 (8.6)	4 (33.3)	8 (66.7)		0 (0)	12 (100.0)	
Age (yr)	33-82 (62.30±0.87)			> 0.999			0.242
< 65	77 (55.4)	14 (18.2)	63 (81.8)		2 (2.6)	75 (97.4)	
≥65	62 (44.6)	11 (17.7)	51 (82.3)		5 (8.1)	57 (91.9)	
Smoking history				0.181			0.086
Non-smoker	27 (19.4)	8 (29.6)	19 (70.4)		3 (11.1)	24 (88.9)	
Light-smoker	19 (13.7)	2 (10.5)	17 (89.5)		2 (10.5)	17 (89.5)	
Smoker	93 (66.9)	15 (16.1)	78 (83.9)		2 (2.2)	91 (97.8)	
TNM stage				0.194			0.335
Ⅰ	25 (18.0)	3 (12.0)	22 (88.0)		1 (4.0)	24 (96.0)	
Ⅱ	27 (19.4)	8 (29.6)	19 (70.4)		2 (7.4)	25 (92.6)	
Ⅲ	38 (27.3)	4 (10.5)	34 (89.5)		0 (0)	38 (100.0)	
Ⅳ	49 (35.3)	10 (20.4)	39 (79.6)		4 (8.2)	45 (91.8)	
Biopsy type				0.132			0.779
Incisional	86 (61.9)	18 (20.9)	68 (79.1)		6 (7.0)	80 (93.0)	
Bronchoscopic	29 (20.9)	2 (6.9)	27 (93.1)		0 (0)	29 (100.0)	
Core biopsy	19 (13.7)	4 (21.1)	15 (78.9)		1 (5.3)	18 (94.7)	
Localisation	3 (2.2)	0 (0)	3 (100.0)		0 (0)	3 (100.0)	
Pleural	1 (0.7)	1 (100.0)	0 (0)		0 (0)	1 (100.0)	
Mediastinum	1 (0.7)	0 (0)	1 (100.0)		0 (0)	1 (100.0)	

### 2.3 *EGFR*与*KRAS*基因突变相关性分析

139例患者中*EGFR*基因突变阳性患者中有1例(4.0%)同时发现*KRAS*基因突变, 而在*EGFR*基因野生型患者中, 有6例(5.3%)发现*KRAS*基因突变, 差异无统计学意义(*P* > 0.05)。

### 2.4 *EGFR*基因突变与靶向治疗疗效

在15例*EGFR*基因敏感突变的患者中, 有3例患者(均为19外显子缺失突变)一线口服TKI药物靶向治疗, 1例部分缓解, 无疾病进展时间3个月; 2例疾病进展, 无疾病进展时间1个月。

## 3 讨论

NSCLC患者治疗前应常规行*EGFR*基因突变检测已成为共识, 但其中仍有争议存在。美国临床肿瘤学会(American Society of Clinical Oncology, ASCO)认为所有NSCLC患者在一线接受EGFR-TKI治疗前都需要进行*EGFR*基因突变状态的检测^[5]^。欧洲肿瘤内科学会(European Society for Medical Oncology, ESMO)建议在不吸烟/轻微吸烟和非鳞癌患者中进行*EGFR*基因突变检测^[6]^。美国病理学家协会、国际肺癌研究协会和美国分子病理学会则建议对含有腺癌成分和不吸烟的患者进行*EGFR*基因突变检测^[7]^。但最新美国国立综合癌症网站(National Comprehensive Cancer Network, NCCN)建议对肺鳞癌尤其是不吸烟、小活检组织或混合型鳞癌应考虑进行*EGFR*基因突变检测^[8]^。这些争议存在的主要原因是目前并没有可靠的临床研究结果证实*EGFR*基因敏感突变的肺鳞癌患者也同肺腺癌患者一样具有明显的EGFR-TKI疗效优势。事实上, 已有临床研究^[9]^发现存在*EGFR*基因敏感突变的肺鳞癌患者, 应用TKI药物治疗疗效并不理想, 但目前仍缺乏大样本的前瞻性临床研究结果。

对于肺鳞癌中*EGFR*突变状态结果的报道也不一致。最近的一项*meta*分析中^[10]^, 334例西欧肺鳞癌患者中*EGFR*基因突变率为3.3%, 474例东亚肺鳞癌患者中*EGFR*基因突变率为4.6%, 均明显低于东亚肺腺癌中50.2%的*EGFR*基因突变率^[11]^。Hata等^[12]^研究更认为, 纯肺鳞癌中无*EGFR*基因突变。但在Lai等^[13]^研究中报道282例肺鳞癌中有41例*EGFR*基因突变, 突变率为14.5%。国内王碧波等^[14]^认为*EGFR*突变确实发生在肺鳞癌患者中。衣素琴等^[15]^在48例肺鳞癌中检测到12例*EGFR*基因突变, 突变率为25%。与其研究结果相似的是, 本研究对139例肺鳞癌进行*EGFR*基因突变检测, 突变率为18%(25/139), 明显高于国外多数文献报道的肺鳞癌*EGFR*基因突变率低于3.6%的研究结果。分析其中原因除了种族差异外, 也可能与本文采用的突变富集液相芯片法敏感性较高有关。之前有研究^[12]^认为, 在纯肺鳞癌中无*EGFR*基因突变, 肺鳞癌中检测到*EGFR*基因突变是因为其中混有腺癌成分, 但在本研究中有86例手术切除的纯肺鳞癌(病理免疫组化染色p40、p63、CK5/6阳性, TTF-1、CK7、CEA阴性), 其中有18例检测到*EGFR*基因突变, 突变率达20.9%, 由此可以看出至少在亚裔人群中, *EGFR*基因突变在肺鳞癌患者中也有发生。在更进一步的亚组分析中发现, 虽然女性和不吸烟的肺鳞癌患者*EGFR*基因突变率高于男性和吸烟患者(33.3% *vs* 16.5%, 29.6% *vs* 16.1%), 但差异无统计学意义(*P* > 0.05, *P* > 0.05), 而年龄、分期、吸烟史及取材方式与*EGFR*基因突变无关(*P* > 0.05)。从这些结果可以看出肺鳞癌*EGFR*基因突变发生特点与肺腺癌不同, 不具备明显的突变优势人群。由于本研究为回顾性研究, 并没有全部观察到*EGFR*基因敏感突变患者口服TKI药物治疗的疗效。在其中检测到*EGFR*基因19外显子敏感突变的9例患者中, 仅有3例患者一线口服TKI药物治疗, 但疗效均不理想, 即使是疗效达到部分缓解的患者疾病稳定的时间也明显短于肺腺癌*EGFR*敏感突变的患者。这似乎提示肺鳞癌中具有敏感突变的*EGFR*基因也许并不是靶向治疗的“驱动基因”, 但这还需大样本的前瞻性临床研究。值得一提的是, 在本研究中也检测到18外显子少见突变3例(2.2%), 20外显子突变6例(4.3%), 这些在肺鳞癌中鲜有报道, 却都高于肺腺癌研究^[11]^报道的结果(1.4%, 2.9%), 其中原因尚不十分清楚, 但也可能从另一方面提示肺鳞癌中*EGFR*基因突变确实与肺腺癌不同。相较之于肺鳞癌中报道不一的*EGFR*基因突变发生率, *KRAS*基因突变就更为罕见, 相关研究报道结果也较少。日本的一项关于非腺癌的研究^[16]^结果显示, 116例肺鳞癌中, 3例*KRAS*基因突变。国内罗炜等^[17]^在454例NSCLC患者*KRAS*基因突变情况分析中发现, 63例肺鳞癌中仅有1例*KRAS*基因突变, 突变率为1.6%。而本研究139例肺鳞癌中, 7例*KRAS*基因突变, 突变率为5.0%, 高于罗炜等^[17]^报道结果, 但与国内衣素琴等^[15]^报道结果相近(4.17%, 2/48)。其中差异可能有地域差异、检测方法不同的原因, 同时也需进一步大样本的临床研究。有研究^[18]^认为, *KRAS*基因突变在吸烟患者中更易发生。但根据一项韩国的报道^[19]^, *KRAS*基因突变与有无吸烟无关。本研究中也发现*KRAS*基因突变与吸烟史无关(*P* > 0.05)。同时在对性别和年龄的亚组分析中也未发现*KRAS*基因突变存在差异。值得一提的是, 与肺腺癌中*KRAS*基因突变不同, 肺鳞癌中*KRAS*基因突变与*EGFR*基因突变不存在明显相关性, 这可能是因为肺鳞癌中不论是*EGFR*还是*KRAS*基因突变率均较低的缘故。

目前尚无任何指南对NSCLC患者的*EGFR*及*KRAS*基因突变检测方法及检测标本做出明确规定。与多数临床研究不同的是, 本研究采用新型的突变富集液相芯片法, 灵敏度可达到0.1%^[20]^, 检测标本不仅包含手术切除的巨大肿瘤组织标本(86/139, 61.9%), 也有支气管镜活检、肺穿刺、胸膜活检等临床更常见的微小组织标本(53/139, 38.1%)。所有标本*EGFR*和*KRAS*基因突变均检测成功, 且检测结果无差异(*P* > 0.05), 甚至我们在血浆游离DNA的基因突变检测中也取得了满意的结果^[21]^。

肺癌的靶向治疗时代已经来临。相比肺腺癌, 肺鳞癌的研究较为滞后, 目前仍无有效的靶向药物指导临床实践。在肺腺癌中获得成功的治疗经验似乎并不能为肺鳞癌患者带来相同的获益。因此, 如何选择肺鳞癌患者的靶向治疗, 还需分子生物研究者和临床肿瘤医师更为深入的探索研究。
